# Genome-wide identification, evolution, and role of SPL gene family in beet (*Beta vulgaris* L.) under cold stress

**DOI:** 10.1186/s12864-024-09995-5

**Published:** 2024-01-23

**Authors:** Guoxing Xue, Weijiao Wu, Yue Fan, Chao Ma, Ruiqi Xiong, Qing Bai, Xin Yao, Wenfeng Weng, Jianping Cheng, Jingjun Ruan

**Affiliations:** 1 College of Agriculture, Guizhou University, 550025 Guiyang, People’s Republic of China; 2 College of Food Science and Engineering, Xinjiang Institute of Technology, 843199 Aksu, People’s Republic of China

**Keywords:** *Beta vulgaris*, *SPL* gene family, Abiotic stress, Gene expression, Genome-wide analysis

## Abstract

**Background:**

SPL transcription factors play vital roles in regulating plant growth, development, and abiotic stress responses. Sugar beet (*Beta vulgaris* L.), one of the world’s main sugar-producing crops, is a major source of edible and industrial sugars for humans. Although the *SPL* gene family has been extensively identified in other species, no reports on the *SPL* gene family in sugar beet are available.

**Results:**

Eight *BvSPL* genes were identified at the whole-genome level and were renamed based on their positions on the chromosome. The gene structure, SBP domain sequences, and phylogenetic relationship with *Arabidopsis* were analyzed for the sugar beet *SPL* gene family. The eight *BvSPL* genes were divided into six groups (II, IV, V, VI, VII, and VIII). Of the *BvSPL* genes, no tandem duplication events were found, but one pair of segmental duplications was present. Multiple *cis*-regulatory elements related to growth and development were identified in the 2000-bp region upstream of the *BvSPL* gene start codon (ATG). Using quantitative real-time polymerase chain reaction (qRT-PCR), the expression profiles of the eight *BvSPL* genes were examined under eight types of abiotic stress and during the maturation stage. BvSPL transcription factors played a vital role in abiotic stress, with *BvSPL3* and *BvSPL6* being particularly noteworthy.

**Conclusion:**

Eight sugar beet *SPL* genes were identified at the whole-genome level. Phylogenetic trees, gene structures, gene duplication events, and expression profiles were investigated. The qRT-PCR analysis indicated that *BvSPLs* play a substantial role in the growth and development of sugar beet, potentially participating in the regulation of root expansion and sugar accumulation.

**Supplementary Information:**

The online version contains supplementary material available at 10.1186/s12864-024-09995-5.

## Background

Sugar beet (*Beta vulgaris* L., 2n = 18) is an economically important crop grown in temperate and cold temperate regions and is the raw material for approximately 30% of the world’s sugar [1]. Sugar beet is also an important raw material for bioethanol and animal feed worldwide [2–3]. According to previous research, sugar beet originated from a halophyte known as *Beta maritima* L [4].. Through artificial domestication and cultivation, sugar beet is mainly used for feed and food, and edible sugar beet is further divided into industrial and edible types [5–9]. Currently, sugar beet is widely grown in Europe and temperate regions, making it an important economic crop [10–11].

Transcription factors play important role in biology [12–13]. Currently, transcription factors such as bHLH [14], MYB [15–16], HSP [17–20], and bZIP [21–22] are widely found in plants and animals. They perform various functions during the growth and developmental stages of organisms to ensure normal growth and development. Therefore, systematic research on biological transcription factors is important. Squamosa Promoter-Binding Protein-Like (SPL) is a plant-specific transcription factor that regulates plant growth and development. The *SPL* gene was first discovered in 1996, and Klein et al. isolated it from the *Antirrhinum majus* inflorescence cDNA library. Because it can recognize and bind to the SQUAMOSA promoter, it was named SBP1 and SBP2 [23]. The SBP domain encoded by the *SPL* gene is highly conserved and contains approximately 76 amino acid residues [23–25]. The SBP domain is divided into three main parts: Zn-1 (Cys-Cys-Cys-His), Zn-2 (Cys-Cys-His-Cys), and a nuclear localization signal (NLS) located at the C-terminal [25–27]. According to the gene structure and phylogenetic tree, 16 *Arabidopsis* SPL family members were identified and divided into eight subgroups (I–VIII) [24, 28]. These *Arabidopsis* SPL family members have been shown to play important roles in the development of *Arabidopsis* stems, leaves, and flowers [29–30]. To date, whole-genome identification and analysis of SPL transcription factors in many plants have been completed, including *Arabidopsis* [24, 28], rice [31, 32], millet [33], quinoa [34], corn [35], tomato [36], buckwheat [37], barley [38], and wheat [39].

SPL transcription factors play vital roles in plant growth and development. For instance, in Switchgrass, when *PvSPL2* expression is suppressed, biomass yield can be enhanced, and the total soluble sugar content can be increased [40]. In *Arabidopsis*, under heat stress, *SPL* genes are downregulated by miR156 to counter the effects of high temperatures [41]. *ZmSPL* in maize regulates several aspects of maize morphology, such as plant height, tillering, and grains [42]. *OsSPL3* in rice can regulate plant cold resistance [43], and *OsSPL14* has been associated with tiller number, grain weight, and disease resistance in rice [44–46]. Many plant *SPL* genes have been discovered and identified, and the functions of some *SPL* genes have been studied. Sugar beet has substantial economic value; however, the *SPL* gene family in sugar beet has not yet been identified. Therefore, it is important to perform genome-wide mining and the systematic identification of *SPL* genes in sugar beet.

Therefore, building upon the sugar beet genome, we systematically excavated, identified, and researched *BvSPL* genes in sugar beet. In this study, we identified eight *BvSPL* genes and analyzed their chromosomal distribution, gene duplication events, *cis*-acting elements, gene structures, and conserved motifs. Moreover, we analyzed the evolutionary relationship between the *SPL* genes of Arabidopsis, rice, maize, buckwheat, sorghum, and tomato and *BvSPL* genes in sugar beet. Additionally, we investigated the expression of *BvSPL* genes under eight forms of non-biological stress in sugar beet seedlings and in different tissues of mature sugar beet, thereby providing a foundation for studying the biological functions of *BvSPL* genes in sugar beet. In summary, a systematic analysis of the sugar beet *SPL* gene family was conducted to identify that *BvSPL* genes have critical roles in the growth and developmental processes of sugar beet. This lays a foundation and provides a reference for future sugar beet research.

## Results

### Identification of sugar beet *SPL* genes

Based on the complete sugar beet genome, two BLAST methods were used to obtain the *SPL* genes. After eliminating duplicate genes, eight *SPL* genes were identified. Depending on the location of the eight *SPL* genes on the nine chromosomes, we named them *BvSPL1*–*BvSPL8*. The biological characteristics of the eight *BvSPLs* were analyzed, including the length of the amino acid sequence, protein molecular weight, protein hydrophilicity, protein isoelectric point, and subcellular localization (Table S1). Subcellular localization prediction revealed that all eight *BvSPL* genes were located in the cell nucleus. The BvSPL protein with the most amino acids had 996 (BvSPL4), whereas that with the fewest amino acids had only 267 (BvSPL3). The isoelectric point ranged from 5.61 (BvSPL4) to 9.63 (BvSPL3), and the protein molecular weight ranged from 30.33 kDa (BvSPL3) to 110.70 kDa (BvSPL4). All BvSPL proteins were found to be hydrophilic.

### Phylogenetic analysis, classification, and multiple sequence alignment of *BvSPL* genes

**Fig. 1:**
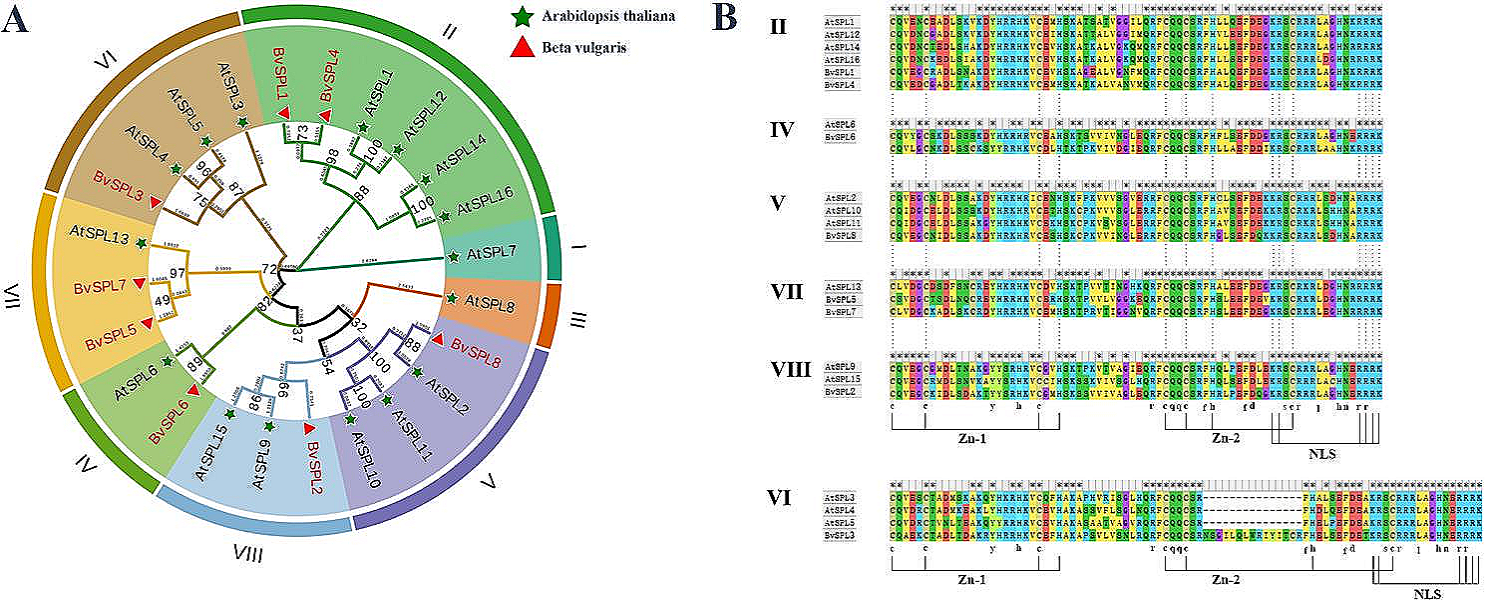
(A) Phylogenetic tree of the relationship between Beta vulgaris and Arabidopsis thaliana SPL proteins. Different block colors represent different subgroups, with green stars representing Arabidopsis thaliana and red triangles representing Beta vulgaris in the legend. (B) Multiple sequence alignment of the SBP domains of different subgroups of Beta vulgaris and Arabidopsis thaliana

To study the evolutionary relationship between sugar beet *SPL* genes, a phylogenetic tree with a bootstrap value of 1000 was constructed using the neighbor-joining (NJ) method, which included eight sugar beet SPL proteins and 16 *Arabidopsis thaliana* SPL proteins (Table S2). Based on the AtSPL classification method [24, 28], the *BvSPL* gene family was divided into six subgroups (II, IV, V, VI, VII, and VIII; Fig. 1A). Compared to the *AtSPL* gene family, the *BvSPL* gene family lacked subgroups I and III. Of these subfamilies, subgroups II and VII each contained two *BvSPL* members, and subgroups IV, V, VI, and VIII each contained one *BvSPL* member. After aligning *AtSPL* and *BvSPL* genes according to their respective subgroups, and based on the characteristics of the SBP domain, the SBP domains of the *BvSPL* genes were obtained (Fig. 1B).

The sequence of the SBP domain of the *BvSPL* gene was approximately 74 amino acids long, of which the CQQC, SCR, and RRR sequences in the SBP domain were highly conserved. All BvSPLs contained two zinc-finger structures (Zn-1 and Zn-2) and a bipartite nuclear localization signal (NLS) motif. However, the Zn-2 (Cys-Cys-His-Cys) sequence of BvSPL3 in subgroup VI had mutated and contained 15 more amino acids than the Zn-2 sequence of the AtSPL protein. Such mutations may cause changes in the zinc finger binding site, thereby affecting protein conformation and endowing the *BvSPL3* gene with new functions. In the other subgroups, Zn-1, Zn-2, and NLS were highly conserved, and the phenomenon that occurred in BvSPL3 in subgroup VI was not observed.

### Analysis of gene structure, motif composition, and *cis*-acting elements of the *BvSPL* gene family

**Fig. 2:**
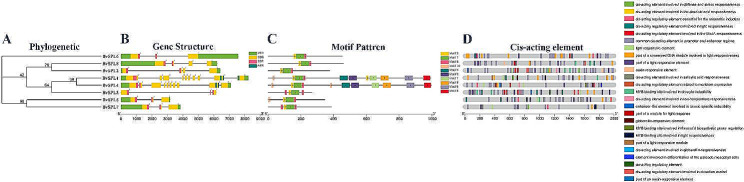
Phylogenetic relationship, gene structure, motif distribution, and cis-acting elements of sugar beet SPL genes. Among them, the number marked on the Node represents the confidence level. (A) Phylogenetic tree of the sugar beet SPL family, each node has 1000 repetitions. (B) Schematic diagram of the gene structure of sugar beet SPL genes, including UTR (untranslated region), CDS (coding sequence), domains (SBP, ANK domains), and introns (Number indicates the phase of the corresponding intron.). Light green represents UTR, yellow represents CDS, pink represents the structural domain SBP, and dark green represents structural domain ANK. (C) Conserved amino acid motifs (motifs 1–10) in BvSPL proteins: the line represents the relative length of the protein. (D) Cis-acting elements in the 2000 bp promoter sequence upstream of the BvSPL genes; different color blocks represent different cis-acting elements

Eight *BvSPL* genes were identified at the genome-wide level; a phylogenetic tree of the full-length sequences of eight BvSPL proteins was constructed; and gene structure, sequence composition, and *cis*-acting elements were analyzed (Fig. 2; Table S3). The intron–exon structures of the same subgroup were similar, but there were large differences between the subfamilies. For example, sub-group II (*BvSPL1* and *BvSPL4*) all had ten exons, while sub-group VII (*BvSPL5* and *BvSPL7*) all had three exons. The other four sub-groups (IV, V, VI, and VIII) had fewer exons, with an average of only 3.5 introns, which was close to sub-group VII. All *BvSPL* genes contained the SBP domain and subgroup II contained both the SBP and ANK domains (Fig. 2B).

To explore the conserved motifs of the eight BvSPL proteins, we used the MEME website to analyze ten conservative motifs of the BvSPL family (Fig. 2C; Table S3). Motifs 1, 2, and 3 were found to exist in the entire BvSPL family, and they were arranged in a specific order of motifs 2, 1, and 3 on the BvSPL protein sequence. However, motifs 4, 5, 6, 7, 8, and 9 only existed in sub-group II, whereas motif 10 only existed in BvSPL5 of sub-groups II and VII.

A similar arrangement of motifs indicates relatively conserved protein structures. All BvSPL proteins had a specific arrangement of motifs 2, 1, and 3, indicating that the BvSPL family was relatively evolutionarily conserved. The arrangement of motifs also supported the reliability of subgrouping the BvSPL family.

To elucidate the function of *BvSPL* genes, the *cis*-acting elements in the 2 kb promoter region upstream of the *BvSPL* genes were investigated (Fig. 2D; Table S3). Of the *cis*-acting elements of *BvSPL*, elements related to light were the most abundant, and all *BvSPL* genes contained these elements. The following elements were related to plant hormones: MeJA (TGACG-motif, CGTCA-motif), abscisic acid (ABRE), salicylic acid (TCA-element), and gibberellin (P-box, GARE-motif). *BvSPL* genes also have *cis*-acting elements related to growth and development, such as *cis*-regulatory elements related to meristem expression and elements involved in defense and stress responses. *BvSPL3* had *cis*-acting elements that responded to low temperatures and *BvSPL3* also had high expression under low-temperature stress. These results indicate that the *BvSPL* gene family plays an important role in plant growth and development.

### Chromosome distribution and gene replication of the *BvSPL* gene

**Fig. 3:**
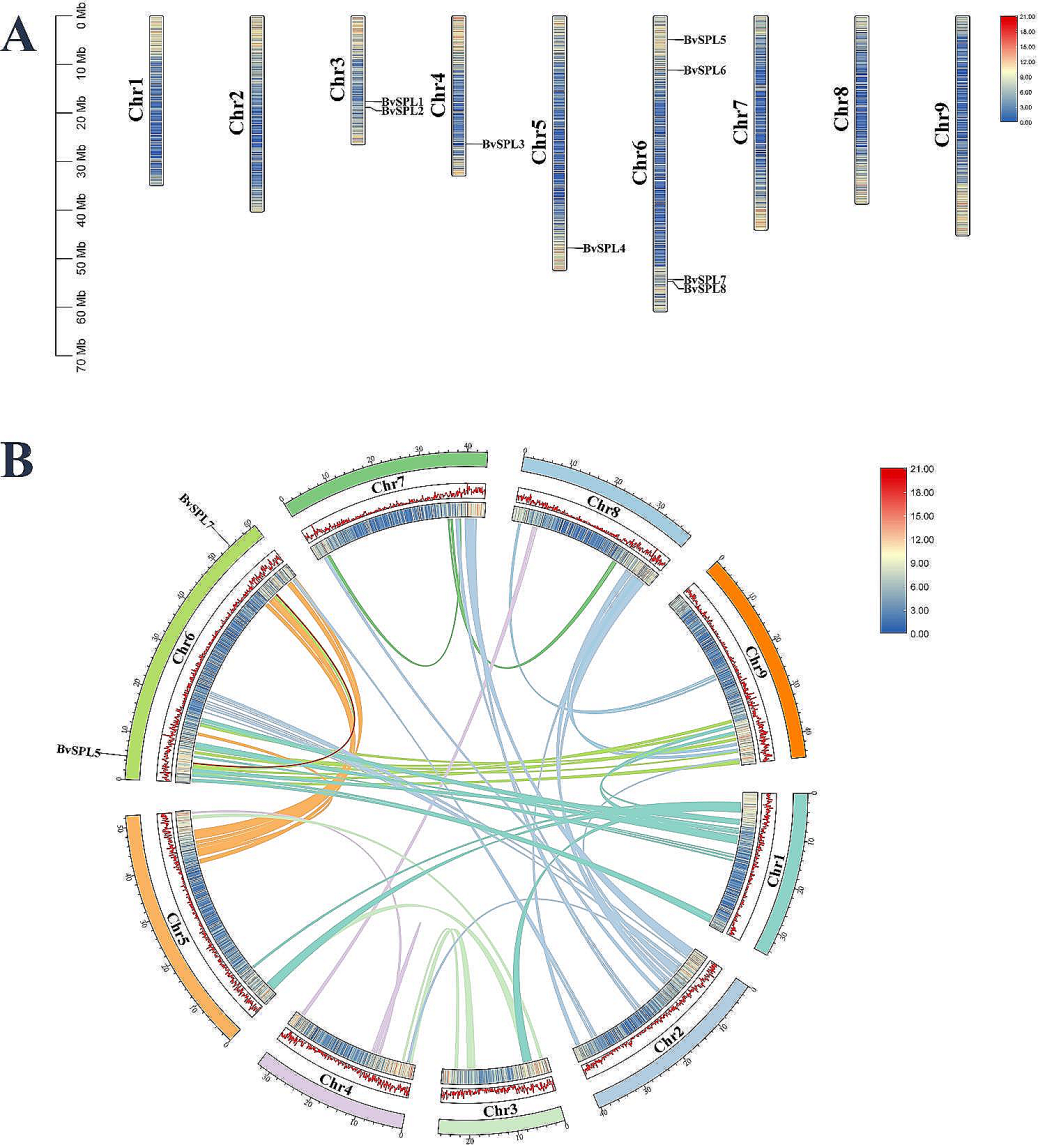
(A) Distribution of the eight BvSPL genes in beet chromosomes, with gene density on chromosomes (Bin size = 100,000). (B) Chromosome distribution and gene duplication relationship of sugar beet SPL genes. The colored lines represent gene pairs between different chromosomes: the red line represents the BvSPL gene pair; from the inside out, the first and second outer circles are chromosome density (Bin size = 100,000), the third is the chromosome; the chromosome color is consistent with the gene pair line color on the chromosome

We determined the physical location map of the eight *BvSPL* genes on the chromosome using the sugar beet genome (Fig. 3A). Eight *BvSPL* genes were unevenly distributed across the nine sugar beet linkage groups (LGs), and the entire *BvSPL* family was distributed on only four chromosomes (Chr3, Chr4, Chr5, and Chr6). Of them, Chr6 had the highest number of *BvSPL* genes (four; ~50%). The least abundant was on Chr4 and Chr5, both of which contained only one *BvSPL* gene (~ 12.5%). The remaining two *BvSPL* genes were found on Chr3 (~ 25%). When one or more identical gene regions appear within 200 kb of a chromosome, it is referred to as a tandem duplication event. However, no tandem duplication events were not observed in the BvSPL family. A segmental duplication event was observed in the *BvSPL* gene family (Fig. 3B; Table S4). This segmental duplication event occurred on Chr6, and the genes involved in the segmental duplication were all members of subfamily VII (*BvSPL5* and *BvSPL7*), which also supports the subgroup grouping of the BvSPL family. Gene duplication events play an indispensable role in the generation of new functions and in gene amplification. The *BvSPL* gene family does not have tandem duplication events; however, there is a pair of segmental duplication events. Therefore, we believe that tandem duplication events have not played a role in the expansion of the *BvSPL* family, while segmental duplication has played a certain role in the expansion of the *BvSPL* family.

### Evolutionary analysis of *BvSPL* and *SPL* genes in different species

**Fig. 4:**
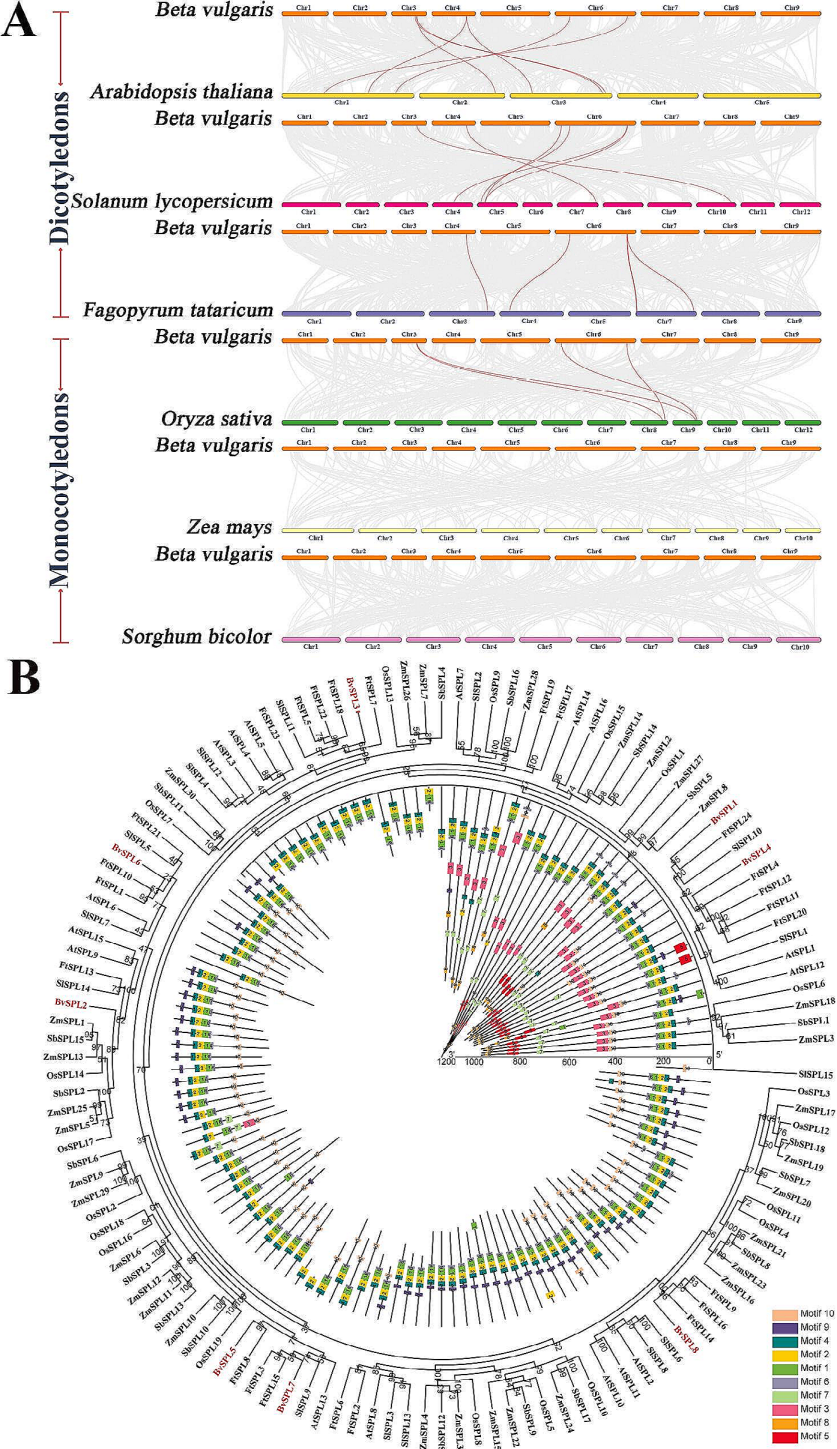
(A) Collinearity analysis of beet with six plants (Arabidopsis thaliana, Solanum lycopersicum, Fagopyrum tataricum, Oryza sativa, Zea mays, and Sorghum bicolor). Red lines represent the species’ beet SPL genes and gene pairs, and gray represents collinear blocks in the beet and the species’ genomes. (B) The phylogenetic tree and motif composition of the SPL proteins of beet and six plants (Arabidopsis thaliana, Solanum lycopersicum, Fagopyrum tataricum, Oryza sativa, Zea mays, and Sorghum bicolor). Different module colors represent different motifs. The numbers on the evolutionary tree represent confidence levels. Red fonts represent BvSPL

To study the evolutionary relationships of the SPL family among different species, a collinearity map (Fig. 4A; Table S5) and a phylogenetic tree (Fig. 4B; Table S6) of *BvSPL* with six species (three dicotyledons: *Arabidopsis thaliana*, *Solanum lycopersicum*, and *Fagopyrum tataricum*, and three monocotyledons: *Oryza sativa*, *Zea mays*, and *Sorghum bicolor*) were constructed. Of the homologous genes of *BvSPL* and *SPL* in the six species (Fig. 4A; Table S5), the homologous pair numbers were *A. thaliana* (seven pairs), *S. lycopersicum* (six pairs), *F. tataricum* (five pairs), *O. sativa* (four pairs), *Z. mays* (zero pairs), and *S. bicolor* (zero pairs). Compared with monocotyledons, *BvSPL* genes have more homologous genes than dicotyledonous plants. This suggests that the *BvSPL* genes may have originated from the ancestors of dicotyledonous plants after the differentiation of monocotyledons and dicotyledons. The *BvSPL3* gene has homologous genes with the three dicotyledonous plants, but not with monocotyledonous plants, indicating that the *BvSPL3* gene may have formed after the differentiation of monocotyledons and dicotyledons.

We constructed a phylogenetic tree of BvSPL proteins and proteins from six other species, and used the MEME website to analyze the protein-conserved motifs of the seven species (Table S6). In the analysis of protein-conserved motifs, motifs 1, 2, 4, and 6 were found in almost all SPL proteins. This suggests that the plant SPL family may have existed before the differentiation of monocotyledons and dicotyledons. Although they evolved in different directions after the differentiation of monocotyledons and dicotyledons, they remained relatively conserved as a whole. From the phylogenetic tree, although *BvSPL* had only five pairs of homologous genes with Tartary buckwheat *SPL*, we found that *BvSPL* was mainly aggregated with Tartary buckwheat *SPL* genes. Therefore, we inferred that the *BvSPL* gene family was closer to the *F. tataricum SPL* gene family.

### Expression patterns of the *BvSPL* gene under different abiotic stresses

**Fig. 5:**
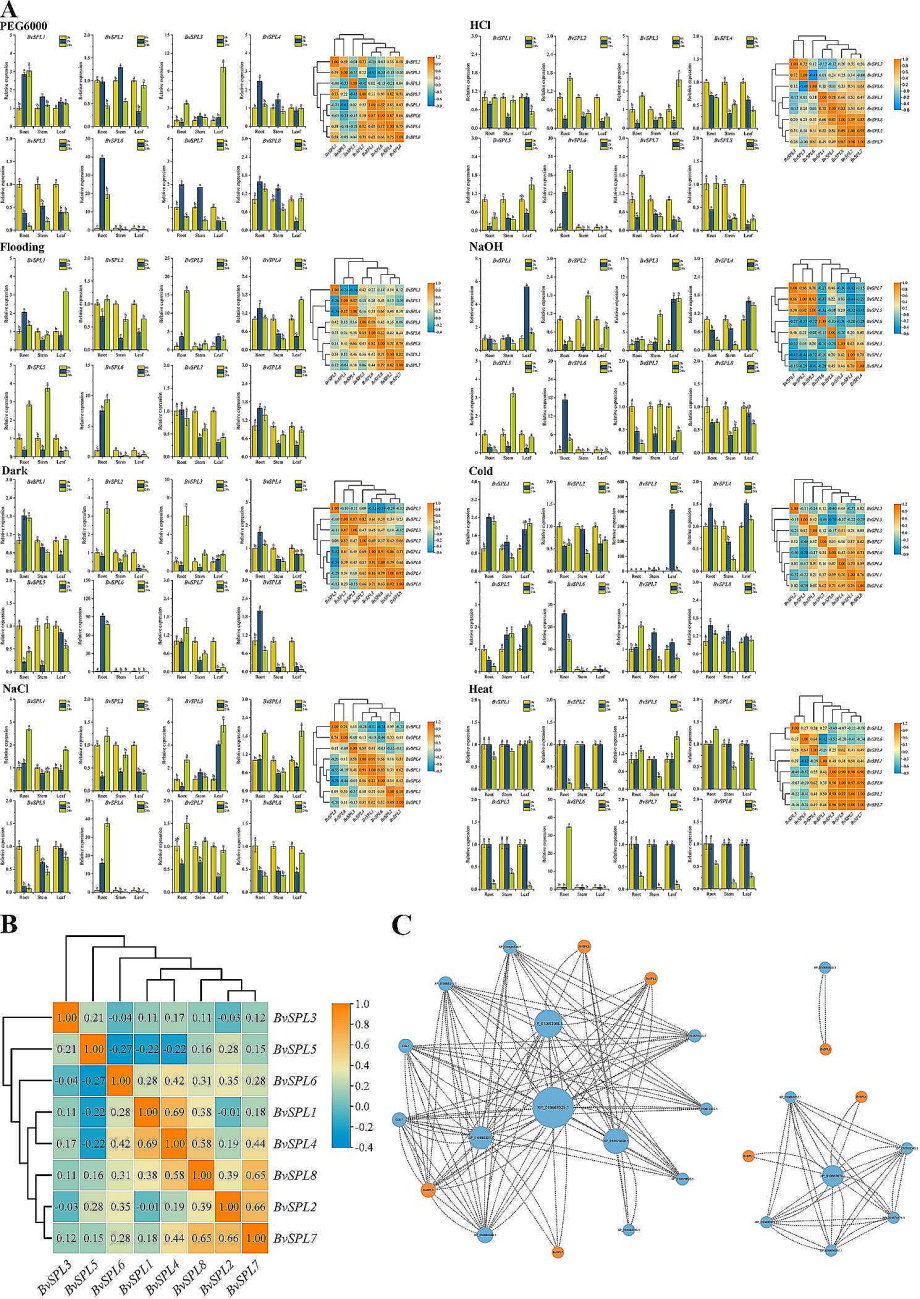
(A) Relative expression levels and gene expression correlations of BvSPL genes in beet seedling roots, stems, and leaves at 0 h, 2 h, and 24 h under eight types of abiotic stress detected using quantitative real-time polymerase chain reaction (qRT-PCR). The lowercase letters above the bars indicate significant treatment differences (α = 0.05, LSD). The expression level of BvSPL gene was normalized to the expression level of BvACTIN, and its relative expression level was displayed at 0 h, 2 and 24 h. (B) Correlation of BvSPL gene expression under eight types of abiotic stress. The expression values of the color gradient mapping from low (blue) to high (red) on the right side of the figure. (C) Predicted protein–protein interaction network of beet BvSPL proteins within the beet. Orange represents BvSPL proteins; blue represents other proteins within the beet; the larger the circle, the more interacting proteins there are

To elucidate the physiological functions of *BvSPL* genes under abiotic stress, qRT-PCR was used to detect gene expression in the roots, stems, and leaves of beet seedlings under eight types of abiotic stresses (PEG, flooding, darkness, salt, acid, alkali, cold, and heat). We found that some genes exhibited marked expression or suppression under abiotic stress and many genes showed related expression under certain types of abiotic stress (Fig. 5A, Fig. S1). For instance, *BvSPL6* was substantially upregulated in the roots under all stress conditions but was downregulated in the stems and leaves. In the roots, the upregulation of *BvSPL6* was extremely high, with most of the differences being more than 20-fold. We also discovered that *BvSPL3* responded to all types of stress and displayed an extremely high, short-term (2 h) response in leaves under cold stress (-4 ℃). Many genes were initially downregulated, followed by upregulation under abiotic stress. For example, under drought stress, the response of *BvSPL2* in the roots, stems, and leaves initially decreased and then increased. Many genes were gradually up-regulated or down-regulated. Under salt (NaCl) stress, the expression of *BvSPL5* in the roots, stems, and leaves gradually decreased. Under alkali stress (NaOH), the expression of *BvSPL3* gradually increased under alkaline stress. Under heat stress (40 ℃), the expression of *BvSPL* genes changed markedly only after 24 h, and was mainly downregulated in roots, stems, and leaves. Therefore, we inferred that *BvSPL* genes are important for resistance to high temperatures.

Using gene heatmaps, the correlations between gene expressions were investigated. The correlation of *BvSPL* gene expression under different types of abiotic stress, and the correlation of gene expression under single stress (Fig. 5A, B, Fig. S1) were investigated. Under alkaline stress (NaOH), positive correlation areas (*BvSPL2*, *BvSPL5*, and *BvSPL7*; *BvSPL1*, *BvSPL3*, and *BvSPL4*) and negative correlation areas (*BvSPL1* and *BvSPL4* with *BvSPL2; BvSPL5*, *BvSPL6*, and *BvSPL7*). We also found positive and negative correlation areas for other types of stress.

We then analyzed the correlation between *BvSPL* gene expression and eight types of abiotic stresses (Fig. 5B). We found that only a few genes had strong correlations, such as *BvSPL2*, *BvSPL7*, and *BvSPL8*. However, most *BvSPL* genes had no strong positive or negative correlations; therefore, we speculated that the correlation of expression among *BvSPL* genes was low.

We predicted the interactions among the eight BvSPL proteins using the STRING online website to speculate on possible protein–protein interactions (Fig. 5C). We found no direct protein–protein interactions among the BvSPL family members, and BvSPL5 did not interact with proteins within the beet. However, BvSPL2, BvSPL7, and BvSPL8 interacted with beet proteins XP_010673830.1, XP_010692327.1, and XP_010693088.1. Therefore, we speculated that BvSPL2, BvSPL7, and BvSPL8 might regulate each other’s expression through three proteins, XP_010673830.1, XP_010692327.1, and XP_010693088.1, as intermediary bridges. This may also be true for BvSPL1 and BvSPL4. This aligns with the gene expression correlation shown in Fig. 5B.

### Expression pattern of *BvSPL* gene in sugar beet maturation

**Fig. 6:**
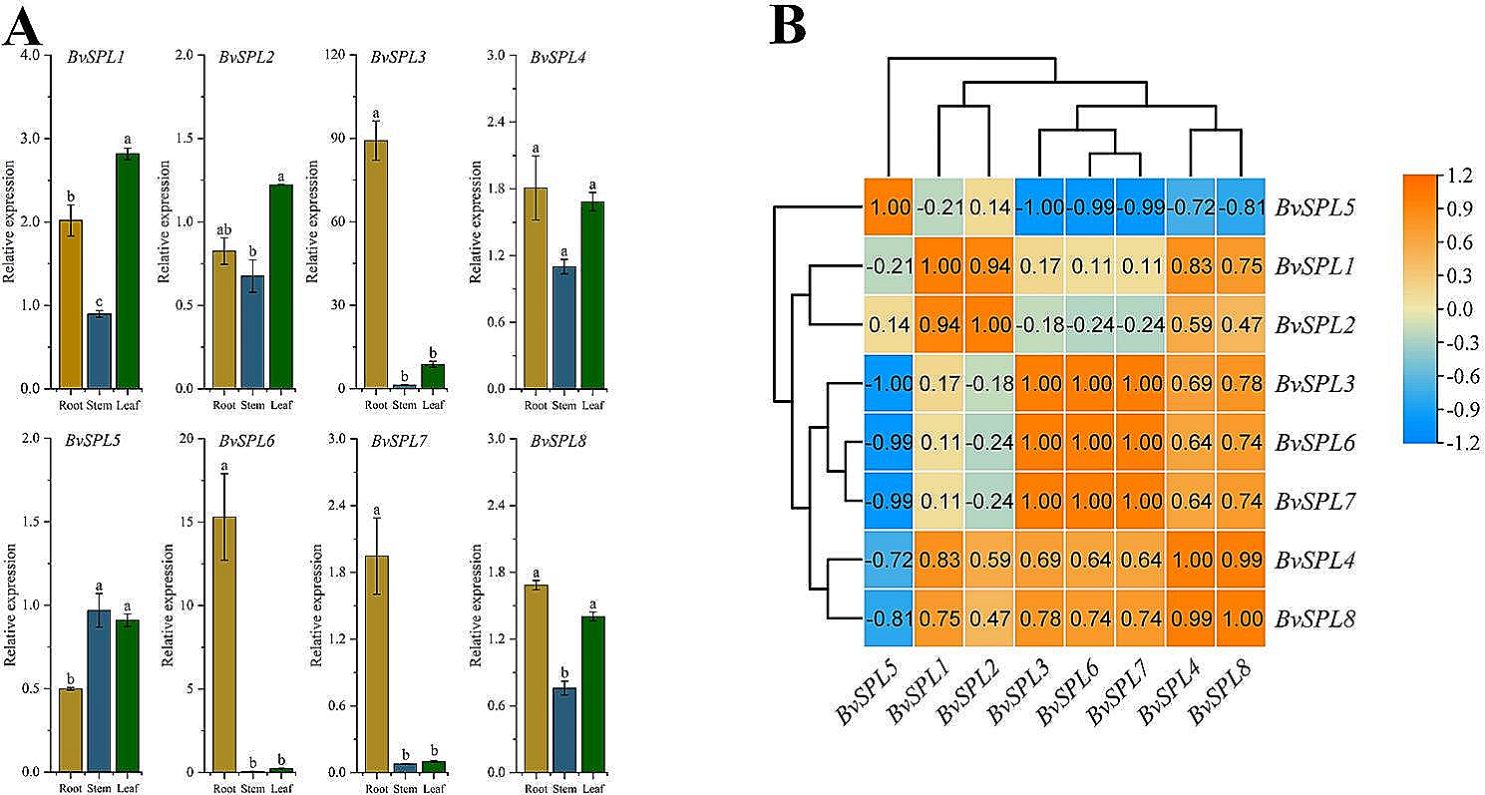
(A) The expression patterns of eight SPL genes in mature sugar beet roots, stems, and leaves were detected using quantitative real-time polymerase chain reaction (qRT-PCR) technology. The lowercase letters above the bars indicate significant differences between treatments (α = 0.05, LSD). The expression level of BvSPL gene was normalized to the expression level of BvACTIN, and its relative expression level was displayed at 0 h, 2 and 24 h. (B) Correlation analysis of the expression of BvSPL genes in mature beet. The expression values of the color gradient mapping from low (blue) to high (red) on the right side of the figure

Beetroots have substantial economic value; therefore, we tested the expression of *BvSPL* genes in the roots, stems, and leaves of mature beet (Fig. 6A, Fig. S1). We found that *BvSPL3* and *BvSPL6* were highly expressed in the roots, and *BvSPL3* was highly expressed in the leaves. However, the expression of *BvSPL7* was significantly down-regulated in both stems and leaves. The expression of *BvSPL5* was down-regulated in roots and *BvSPL8* in stems. All these suggest that the sugar beet SPL family plays an important role in the maturation of sugar beet.

In the correlation analysis (Fig. 6B), clear positive and negative areas of correlation were found. There were seven genes in the positively correlated area and one gene (*BvSPL5*) in the negatively correlated area. We found that *BvSPL5* was negatively correlated with seven other genes except for *BvSPL2*. *BvSPL3*, *BvSPL6*, and *BvSPL7* had a correlation of unity and were all expressed at much higher levels in beetroots than in stems and leaves. However, *BvSPL3* was highly expressed in both roots and leaves, whereas *BvSPL6* and *BvSPL7* were extremely low in stems and leaves, but higher in roots. Therefore, we believe that there is a coupling phenomenon in the correlation between *BvSPL3*, *BvSPL6* and *BvSPL7*.

## Discussion

### Evolution of SPL gene family in sugar beet

SPL transcription factors in plants are involved in important physiological processes such as plant growth, metabolism, gibberellin signal transduction, and leaf formation [47]. In the present study, the beet SPL transcription factor family was systematically analyzed and the functions of eight *BvSPL* genes were explored. Analysis of the physicochemical properties of the eight *BvSPL* genes revealed that the physicochemical properties of *BvSPL3* differed substantially from those of the other seven *BvSPL* genes. This indicates that the grand average hydrophobicity of the BvSPL3 protein was − 1.24, which was the lowest in the beet SPL family. For the Instability Index, the BvSPL3 protein (49.05) was also the lowest. The molecular weight (~ 30.3 kDa) and length (267) of BvSPL3 were the lowest, whereas its isoelectric point (9.63) was the highest. In the entire beet SPL family, BvSPL3 is closer to BvSPL1 and BvSPL4, which are more closely related in terms of evolution; however, its physicochemical information is opposite to that of BvSPL1 and BvSPL4. In the expansion and contraction of beet *SPL* gene family members, tandem duplication did not play a role in the amplification of the *BvSPL* family, whereas segment duplication played a role. In the multi-sequence alignment, the Zn-2 region of the *BvSPL3* gene has 15 more amino acids than the other *BvSPL* genes. In the evolutionary analysis of the SPL transcription factor family of dicotyledonous plants, it was found that the *BvSPL3* gene had genes homologous to three dicotyledonous plant *SPL* genes, indicating that the *BvSPL3* gene may be a relatively primitive gene. However, the *BvSPL3* gene may be a special *SPL* gene that has evolved in beet to adapt to survival and has undergone gene sequence changes. Therefore, we speculate that the *BvSPL3* gene plays an extremely important role in the growth and development of beet and may be related to the swelling of beetroot.

In the evolution of the sugar beet SPL transcription factor family, three monocotyledonous plants (*O. sativa* [31–32], *Z. mays* [35], and *S. bicolor* [35]) and three dicotyledonous plants (*A. thaliana* [24–28], *S. lycopersicum* [36], and *F. titanium* [37]) were selected for a comparative analysis of the SPL transcription factor families (Fig. 4). Phylogenetically, sugar beet SPL members clustered more with buckwheat and tomatoes, a finding that is consistent with that of Dohm et al. [1].. In the analysis of the conserved sequences of sugar beet SPL and those of the six species, most SPL proteins shared highly similar conserved sequences, including sequences 1, 2, 4, and 6. In the collinearity analysis, sugar beet *SPL* genes had more homologous genes with dicotyledons. *BvSPL3* and *BvSPL8* had homologous genes in the three dicotyledons but not in monocotyledons. This suggests that these three genes may be unique genes that evolved in dicotyledons after the differentiation of monocots and dicots.

Research has suggested that introns can produce different exon combinations through selective splicing during protein translation, thus translating different proteins and increasing their complexity [48, 49]. Furthermore, introns have been found to function independently of their coding genes. For example, introns can regulate cell starvation resistance through the TORC1 and PKA nutrient-sensing pathways [50]. Analysis of the beet SPL transcription factor family revealed that subfamily II had far more introns than the other subfamilies. Most researchers believe that in the ancient ancestors of organisms, there were a large number of introns, but with the evolution of organisms, a large number of introns were lost, which is the early intron hypothesis [51–54]. Therefore, we speculate that SPL subfamily II is older than the other subfamilies.

### Response of beet *SPL* gene to abiotic stress and its spatiotemporal expression in different tissues

In organisms, gene expression is often a prerequisite for gene function, and gene expression patterns are usually related to gene function [55]. The *SPL* gene is widely involved in plant growth and development and plays a vital role in promoting the transition of plants from the seedling stage to the mature stage [56, 57]. For example, in *A. thaliana*, *AtSPL3*, *AtSPL4*, and *AtSPL5* genes are involved in morphogenesis [30]. We examined the gene expression of all members of the beet SPL family under eight abiotic stress conditions (Fig. 5, Fig. S1). The results showed high expression or repression of *BvSPL* genes in abiotic stress, such as *BvSPL1*, *BvSPL3*, *BvSPL5*, and *BvSPL6*. In abiotic stress, there was a phenomenon where the gene expression of *BvSPL* genes first increased and then decreased, or decreased and then increased. For instance, under alkaline stress (0.2 mol/L NaOH), *BvSPL2* showed a decrease in expression, followed by an increase in expression across the roots, stems, and leaves, similar to *BvSPL5* in the stems and leaves. Under drought stress (PEG6000), *BvSPL4*, *BvSPL7*, and *BvSPL8* showed an increase, followed by a decrease in the roots and stems. Similar phenomena were observed under other stress conditions. This suggests that these genes may be fast-responding and capable of helping beet resist damage caused by adverse conditions in the short term. Similar phenomena have been observed in other species [33, 58].

Sugar beet can withstand a variety of abiotic stresses, such as salinity, drought, cold, and heat [59–64]. Therefore, we speculate that the *SPL* gene family plays an important role in abiotic stress resistance in sugar beet. *BvSPL3* and *BvSPL6* may play strong roles in helping sugar beet resist abiotic stress. Under eight types of stress conditions, the *BvSPL6* gene was highly expressed in the roots, whereas its expression was suppressed in the stems and leaves. This indicates that the *BvSPL6* gene might be a key gene in sugar beet for controlling plant stress resistance, with sugar beet resisting abiotic stress either directly through the expression of *BvSPL6* or by regulating the expression of other genes through *BvSPL6*. Whether *BvSPL6* assists sugar beet in resisting abiotic stress through direct expression or acts as a messenger to help sugar beet resist abiotic stress by indirectly regulating other genes, *BvSPL6* is a key gene in sugar beet resisting abiotic stress and is worth investigating.

*BvSPL3* exhibited extremely high gene expression in leaves under cold stress. Cold temperatures are critical factors limiting the economic yield of sugar beet, particularly long-term cold temperatures during the seedling growth phase, which can ultimately lead to slow root growth and reduced sugar output [64, 65]. Cold temperatures can also lead to a decrease in photosynthetic efficiency, CO_2_ assimilation rate, and leaf transpiration rate in sugar beet seedlings, thereby affecting sugar beet growth and development [66, 67]. Therefore, we hypothesized that *BvSPL3* may help sugar beet alleviate these situations. Under heat stress, most sugar beet *SPL* genes show gene expression suppression in sugar beet roots, stems, and leaves, indicating that the sugar beet SPL family is of significant importance to the heat tolerance of sugar beet.

In this study, we examined the expression levels of *BvSPL* genes in mature sugar beet (Fig. 5, Fig. S1). *BvSPL3* and *BvSPL6* were highly expressed in the roots, with *BvSPL3* showing a differential expression up to 90 times. The sugar beetroot is where sugar accumulates and is also the most important economic value of sugar beet. In sugar beet, BvCPD was found to promote the development of thin-walled cells and vascular bundles of the main root of sugar beet, thereby affecting the growth and development of the main root of sugar beet, ultimately affecting the size of the main root of sugar beet [68]. The protein encoded by *BvTST2.1* in sugar beet is a sucrose-specific transport protein, which is responsible for the absorption of sucrose in the vacuoles of the main root of sugar beet. Its expression promotes sucrose accumulation in sugar beet roots [69]. The high expression of *BvSPL3* and *BvSPL6* in the main roots of mature sugar beet suggests that they may influence the size of the main root and sugar accumulation. In conclusion, *BvSPL3* and *BvSPL6* warrant further investigation.

## Conclusion

This is the first study to analyze the *SPL* gene family in sugar beet on a whole-genome scale. Based on the whole genome, eight members of the sugar beet *SPL* gene family were identified and the gene structure, conservative motifs, subfamily grouping, evolutionary relationships, abiotic stress, and spatio-temporal expression patterns of the eight *BvSPL* genes were analyzed, thereby inferring their possible biological functions. In evolution, fragment duplication of the sugar beet *SPL* family has played a certain role in the expansion of the sugar beet *SPL* family. The *BvSPL6* gene was highly expressed in roots under eight abiotic stresses, and also in roots at maturity. *BvSPL3* may also have important biological functions in sugar beet resistance to cold stress. In the mature period, *BvSPL3* may play a certain role in sugar beet enlargement or sugar accumulation.

## Materials and methods

### Sugar beet material and abiotic stress

The *B. vulgaris* variety (2n = 18) MA097 (Denmark Mairuibo International Seed Industry Co., Ltd., The Harbin Representative Office, Harbin, China) used in this experiment was provided by Professor Ruan Jingjun. Plant materials were grown in an artificial climate room at the College of Agriculture, Guizhou University. After waiting for the sugar beet to mature, samples of the root, stem, and leaves from beet with good growth status and similar morphology (five replicates) were taken and immediately stored at -80 °C. When sugar beet seedlings grown in the same artificial climate room reached 21 d, they were subjected to abiotic stress (acid: 0.2 mol/L HCl, alkali: 0.2 mol/L NaOH, salt: 5% NaCl, drought: 30% PEG6000, flooding: submerging the entire plant, darkness: complete darkness, heat: 40 °C, cold: 4 °C). For the acid, alkali, salt, and drought treatments, the roots were submerged in solutions of the same volume. After 0 h, 2 h, and 24 h of treatment, samples of the root, stem, and leaves (five replicates) were taken and immediately stored at -80 °C.

### Whole genome identification of sugar beet *BvSPL* genes

The sugar beet genome was downloaded from the genome website (https://plants.ensembl.org/data/ftp/index.html) and gene and amino acid sequences were extracted. SPL gene information was obtained from Arabidopsis (https://www.Arabidopsis.org/) and rice (http://Rice) and potential sugar beet SPL proteins from *Arabidopsis* and rice SPL amino acid sequences were identified using BLASTp (score ≥ 100, e value ≤ 1e − 10).

Next, we obtained a Hidden Markov Model (HMM) consistent with the SPL structural domain (PF03110) from the Pfam protein family database (htxxp://pfam.sanger.ac.uk/) and used HMMER3.3.2 software (default parameters) (htxxp://HMMER.org/) to search for SPL proteins. All possible *BvSPL* genes used were SMART (htxxp://smart.embl-heidelberg.de/) and CD-Search (https://www.ncbi.nlm.nih.gov/Structure/cdd/cdd.shtml) was used to confirm the structural domains of all possible BvSPL proteins to finally obtain the *BvSPL* genes.

The protein-coding sequence length, molecular weight (MW), isoelectric point (pI), subcellular localization, grand average hydrophobicity, and instability index (II) of the BvSPL genes were determined. Subcellular localization was obtained from the WoLF PSORT website (htxxps://psort.hgc.jp/), MW and PI were obtained from the ExPASy website (htxxp://web.expasy.org/protparam/), and the Grand Average of Hydropathicity and Instability Index (II) was obtained using TBtools software (htxxps://github.com/CJ-Chen/TBtools).

### RNA extraction, cDNA reverse transcription, and qRT-PCR analysis of total material

RNA was extracted using a plant RNA extraction kit (Takara Biomedical Technology Co., Ltd., Beijing, China). The concentration and purity of total RNA were measured using a spectrophotometer and reverse transcribed into cDNA using the Hiscript II Q RT Supermix for qPCR kit (Vazyme Biotech Co, Ltd., Nanjing, China). The qRT-PCR primers were designed using Primer Premier 5.0. The internal reference gene was *BvACTIN* [70–71]. The qRT-PCR was performed using SYBR Premix Ex Taq II (Takara Biomedical Technology Co., Ltd., Beijing, China) and repeated at least three times. The relative gene expression was calculated using the 2^−(ΔΔCt)^ method.

### *BvSPL* gene structure, *cis*-acting elements, conserved motifs, and protein interactions

The TBtools software was used to align *BvSPL* genes with sugar beet genes and to construct a *BvSPL* gene map. The PlantCare website (http://bioinformatics.psb.ugent.be/webtools/plantcare/html/) was used to predict the possible *cis*-acting elements (upstream of 2000 bp) in the promoter of the *BvSPL* gene family. The MEME website (htxxps://meme-suite.org/meme/tools/MEME) was used to analyze the ten most conserved motifs in the full-length protein sequence of the *BvSPL* family. MEGA11 software was used to align the SPL protein structural domains based on different subgroups of sugar beet and *Arabidopsis* using the default ClustalW parameters. The STRING website (htxxps://cn.string-db.org/) was used to predict potential protein interactions within the sugar beet BvSPL family, and the results were visualized using the Cytoscape software.

### Chromosomal distribution and gene duplication of *BvSPL* genes

The physical location information of *BvSPL* genes and the gene density information of the chromosomes were extracted from the sugar beet genome and plotted. The MCScan X package was used to analyze gene duplication events of the BvSPL genes (default parameters), and TBtools software (https://github.com/CJ-Chen/TBtools) was used for homology analysis and plotting.

### Statistical analysis

JMP software (version 6.0) was used for the analysis of variance (ANOVA) and conducted Least Significant Difference (LSD) comparisons at a significance level of 0.05 (*p* < 0.05). Origin software was used to plot the histograms of gene expression levels.

### Electronic supplementary material

Below is the link to the electronic supplementary material.

Supplementary Material 1

Supplementary Material 2

Supplementary Material 3

Supplementary Material 4

Supplementary Material 5

Supplementary Material 6

Supplementary Material 7

Supplementary Material 8

## Data Availability

The entire *Beta vulgaris* genome sequence information was obtained from the Ensembl Genomes website (http://ensemblgenomes.org/). *B. vulgaris* variety (2n = 18) MA097 used in the experiment was supplied by Prof. Jingjun Ruan of Guizhou University. The datasets supporting the conclusions of this study are included in the article and its additional files.

## Abbreviations

qRT-PCR: Quantitative real-time polymerase chain reaction
SPL: Squamosa Promoter-Binding Protein-Like
NLS: Nuclear localization signal
PEG: Polyethylene glycol
TORC: Transducer complexes of activity-regulated
PKA: Protein kinase
HMM: Hidden Markov Model
MW: Molecular weight
pI: Isoelectric point
ANOVA: Analysis of variance
LSD: Least Significant Difference
