# Supplementary material for: Genome-wide identification, evolution, and role of SPL gene family in beet (Beta vulgaris L.) under cold stress
Source: BMC Genomics. 2024 Jan 23;25:101. doi: 10.1186/s12864-024-09995-5 (PMC10804631; doi:10.1186/s12864-024-09995-5)

Figures. S1: qPCR gene dissolution curves of eight *BvSPL* genes and internal reference gene *BvACTIN*.


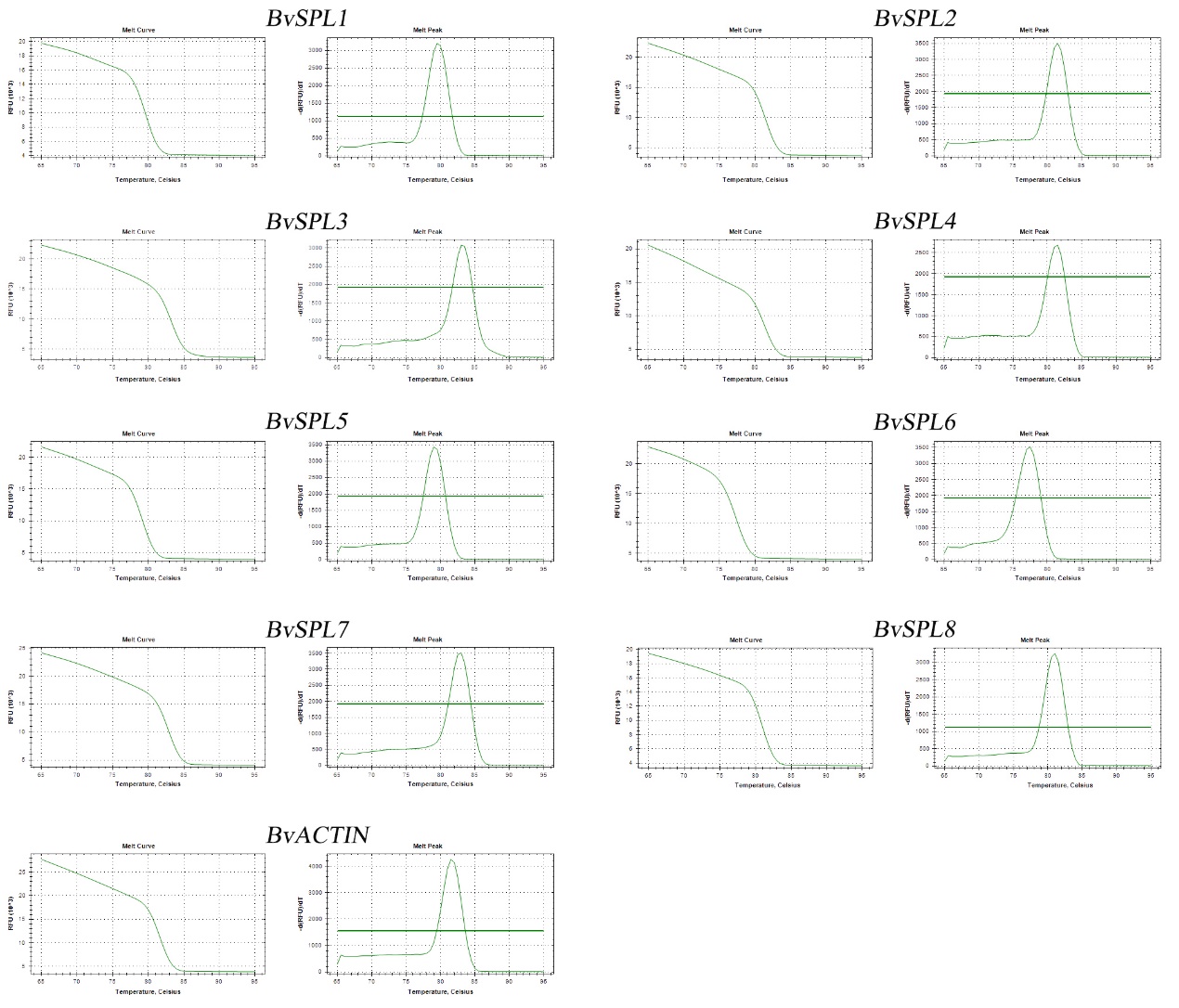

Supplement: Supplementary file 8 — Supplementary Material 8 [file 12864_2024_9995_MOESM8_ESM.docx]
